# WHAT'S MOTIVATION GOT TO DO WITH IT? USING LATENT PROFILE ANALYSIS FOR BIOMARKERS & PHYSICAL ACTIVITY IN OLDER ADULTS

**DOI:** 10.1093/geroni/igz038.3384

**Published:** 2019-11-08

**Authors:** Elizabeth Teas, Jay Kimiecik, Rose Marie Ward, Kyle Timmerman

**Affiliations:** 1 Purdue University, West Lafayette, Indiana, United States; 2 Miami University, Oxford, Ohio, United States

## Abstract

Heart disease is prevalent among older adults. The aim of this study was to a) identify different health behavioral motivation profiles among older adults; and b) investigate if these profiles differed in physical activity and cardiometabolic risk factors. Data on 79 participants (mean age = 68.76 years) was collected. Participants’ degree of intrinsic/extrinsic motivation for diet and exercise was assessed using intuitive eating and self-determination scales. Cardiometabolic risk factors included inflammation and blood lipids. Latent profile analysis was used to identify the optimal number of groups and one-way ANOVAs assessed group differences on the variables of interest. Three profiles were found to best represent the data. The most self-determined, or most intrinsically motivated, group comprised the highest number of participants. In line with Self-Determination Theory, this group demonstrated the highest levels of objective and self-reported physical activity as well as the lowest inflammation and most optimal cholesterol measures. The group with the lowest intuitive eating and high identified exercise regulation scores exhibited the worst outcomes among the three groups. The results suggest that among older adults, different types and levels of motivation for diet and exercise can coexist and interact, and these differences produce varying health outcomes. If supported by future work, these findings can inform practitioners in developing more specific and tailored interventions relevant to older adults based on their motivational profile.

